# Round the Hospitals

**Published:** 1917-10-13

**Authors:** 


### ROUND THE HOSPITALS.

The meeting of the College of Nursing, Limited, in the University College building at Nottingham on Monday last, at which Miss McCall Anderson, R.R.C., late matron of St. George's Hospital, London, presided, afforded the encouraging evidence provided by a large and representative audience of nurses, that the College of Nursing is progressing steadily in the favour and support of the profession. Such an audience has a special significance at the moment, which was emphasised by the presence amongst others of Miss Knight, matron of the General Hospital, Nottingham; Miss Sparshott, R.R.C., matron of the Royal Infirmary, Manchester; Miss Dwight, matron of Bagthorpe Infirmary, Nottingham; Miss Smith, the Cedars Convalescent Hospital; Miss Taylor, Basford Sanatorium; Miss Berry, matron, Chesterfield Hospital; and Miss Ross, inspector, Queen Victoria Jubilee Institute.

Miss Rundle, secretary of the College, was the principal speaker, and at the outset explained fully the objects and aims of the College in a clear and instructive address to a most appreciative audience. Speaking generally, she said, nothing had emphasised the recognised value of the College amongst all shades of opinion inside and outside of the profession so much as the claims which had arisen for representation on the first Council of the College. They must bear in mind that the College was a voluntary body of trained nurses, and that it was for the nurses to manage their own College. In due course a body of professional women who became members of the College would seek statutory powers under an Act of Parliament, when it would be necessary to enlarge the College Council, to admit representatives of Government bodies, and of the medical profession and others directly interested in the training of nurses, both in voluntary hospitals and Poor-Law infirmaries. In this connection it must be continuously borne in mind that two-thirds of the whole College Council would be elected by the nurses on the Register.

Proceeding, Miss Rundle spoke as follows:—

The question might be asked why this representative Council had not been already formed and the Bill presented to Parliament. Why was there a delay? The answer is that more than a year ago another society, the Royal British Nurses' Association, approached the College of Nursing with a view to affiliation, as the objects of the two institutions seemed similar. To make this affiliation possible, a Supplemental Charter to that already possessed by the Royal British Nurses' Association was necessary to be approved by the Privy Council, and it was agreed that its members should be entitled to membership and the benefits of the College without a further fee. While the Royal British Nurses' Association had been conducting this business with the Privy Council and the amalgamation was not complete, the College had felt it was a point of honour to refrain from any policy of development on any lines whatever until these two bodies were represented in a United Council. If this amalgamation was not effected, the programme of the College would continue as hitherto. It was felt by the Council that its policy would be shortsighted if it were not willing to promote in the profession a spirit of unity—for unity is strength. At the same time, it could not be effected without a spirit of give and take, and it must be realised that an old conservative Association might be fearful lest its identity should be lost in a young growing institution, however well safeguarded. A true healthy spirit in a community, however, is one which seeks the greatest good for the greatest number.

Miss Rundle reminded the nurses of the value of such an organisation as that represented by the College of Nursing. It was a pleasure to her to be able to state that she was already in a position to tell them of more than one hospital committee which had thought it worth while to pass special resolutions affecting the conditions of training in their institution, so that their nurses should be qualified for membership of the College in the future.

**ROUND THE HOSPITALS—** (*concluded*).

At the conclusion of Miss Rundle's address, a great many questions were asked and answered, all present showing a keen interest in the many points raised. Great satisfaction was expressed at the information obtained. Indeed, there seemed to be a natural wish, on the part of the nurses present, to hasten the time when, by joining the College Register, they would be entitled to claim that they had become a part of the great organisation represented by the College of Nursing. The arrangements for the meeting were excellent, and the sincere and hearty thanks of all interested in the College are due to those members of it who made the arrangements and invited Miss Rundle to address the meeting. The more these meetings are multiplied up and down the country, and the sooner they are held, the better it will be for British nurses and the nation who owe them so much.

---

The anniversary of Nurse Cavell's death, October 12, will be commemorated not only in this country but in France, and probably other countries too. To mark the anniversary of the execution of Miss Edith Cavell by the Germans, M. Justin Godart, Under-Secretary of State for the Medical Services in France, will, on October 12, 1917, unveil a bust in Edith Cavell's memory in the hospital and school of nursing in Paris which bears her name. On the same day a lecture is being given at the Æolian Hall, New Bond Street, London, at 3.30 p.m., by M. Gaston de Laval, which the American Ambassador promised to attend, and Miss Edith Evans to sing. Tickets could be obtained from the organising secretary of the Edith Cavell Homes of Rest for Nurses, 25 Victoria Street, S.W. 1. On Thursday afternoon, the 11th inst., a concert took place at the Mansion House on behalf of the Edith Cavell Homes of Rest for Nurses. Many nurses who have already enjoyed a pleasant holiday at the Cavell Home at Little Wych, near Bridport, have expressed their gratitude. We are glad to be able to announce that Coombe Head, the Cavell Home at Haslemere, with accommodation to receive nine nurses per month in a beautiful country home, will be open for their reception about the 16th inst.

---

On May 12 last, page 120, we gave an account of a number of presentations made to Miss Herbert, the late matron, on relinquishing her post at Worcester General Infirmary. To these must now be added a cheque for £116 18s., accompanied by an illuminated roll of the subscribers, presented to Miss Herbert by the Mayoress of Worcester at a meeting held at the infirmary on Tuesday, the 2nd instant, which was remarkable in the history of such meetings on account of the large and representative character of the attendance. The speeches showed the high esteem in which Miss Herbert was deservedly held, and the void created by her departure from the city of Worcester. The Dean, the Canons, the chairman and women governors of Worcester Infirmary all combined to praise and extol the value of Miss Herbert's services, and the sense of emulation in recognition, which characterised the proceedings, called forth some remarkable speeches. Miss Herbert modestly transferred the praise, or as much of it as possible, to the staff, the members of the Ladies' Guild, and Mrs. Ernest Day, who, in response, testified that the subscriptions, which collectively represented the purse of money, had come as spontaneous gifts from all sides, and especially from the nurses whom Miss Herbert had trained. Worcester has the reputation of being severely economical in war-time, which may account for the welcome absence of the usual tea on an occasion, which may prove memorable in the history of the institution.

---

We are glad to note that a new form is being taken in the case of testimonials to matrons, which shows a tendency to recognise artistic value and practical utility. Miss C. Tisdell, R.R.C., after two and a half years as matron at Stoke War Hospital, which she is leaving for new work in London, being known as a collector of china, has had presented to her a handsome and highly finished example of artistic workmanship in the form of a china cabinet. In the hospital Orders of the Day we find the following: "The Administrator desires to place on record his sense of regret, shared by all the medical, nursing, and general staff, as well as by the patients of the War Hospital, at the resignation of the matron, Miss Tisdell, and of the loss to the institution of her valuable services. Under her capable mangement, supervision, and conciliatory manner, an efficiency has been obtained which has been evident in all directions."

---

Military matrons have experiences which may linger in their memory. Miss Lilian Birkin, who has received the Order of the British Empire, was welcomed back from Buckingham Palace by the soldier patients at the Bayley Hospital, Nottingham, who sang "To-night's the Night." As she left her taxi at the entrance to the hospital she encountered a guard of honour, consisting of the soldiers in residence, and made her way to the hospital entrance through an archway formed by crossed crutches.

---

**☛ It is our invariable practice to pay for all items of news which we may publish. The minimum payment is 5s. Paragraphs of about twenty lines in length—that is to say, of 150 words or less—are preferred. In this section during the war we propose to give 10s. to the correspondent who may send us in any week the best incident connected with hospital life and work. In this way we hope to afford to all practical aid and encouragement; while those who reciprocate will be able to render us invaluable service. Contributions should be addressed to The Editor, The Hospital, 28 and 29 Southampton Street, Strand, London, W.C. 2, and, to be in time for the current week's issue, should reach him by the first post on Mondays.**

For Nursing Affairs in Ireland, see p. 34; Matrons' and Sisters' Appointments, p. 40.

